# A novel multinuclear solid-state NMR approach for the characterization of kidney stones

**DOI:** 10.5194/mr-2-653-2021

**Published:** 2021-08-20

**Authors:** César Leroy, Laure Bonhomme-Coury, Christel Gervais, Frederik Tielens, Florence Babonneau, Michel Daudon, Dominique Bazin, Emmanuel Letavernier, Danielle Laurencin, Dinu Iuga, John V. Hanna, Mark E. Smith, Christian Bonhomme

**Affiliations:** 1 Laboratoire Chimie de la Matière Condensée de Paris, LCMCP, Sorbonne Université, CNRS, 75005 Paris, France; 2 AP-HP, Hôpital Tenon, Explorations Fonctionnelles Multidisciplinaires et INSERM UMRS 1155, Sorbonne Université, Hôpital Tenon, Paris, France; 3 Institut de Chimie Physique, UMR CNRS 8000, Bâtiment 350, Université Paris Saclay, 91405 Orsay CEDEX, France; 4 Laboratoire de Physique des Solides, UMR CNRS 8502, Bâtiment 510, Université Paris-Sud, 91405 Orsay CEDEX, France; 5 Institut Charles Gerhardt Montpellier, CNRS, ENSCM, Université de Montpellier, Montpellier, France; 6 Department of Physics, University of Warwick, Gibbet Hill Road, Coventry CV4 7AL, United Kingdom; 7 Department of Chemistry, University of Southampton, Southampton SO17 1BJ, United Kingdom; 8 General Chemistry (ALGC) – Materials Modelling Group, Vrije Universiteit Brussel (Free University Brussels – VUB), Pleinlaan 2, 1050 Brussels, Belgium

## Abstract

The spectroscopic study of pathological calcifications (including kidney stones) is extremely rich and helps to improve the understanding of the physical and chemical processes associated with their formation. While Fourier transform infrared (FTIR) imaging and optical/electron microscopies are routine techniques in hospitals, there has been a dearth of solid-state NMR studies introduced into this area of medical research, probably due to the scarcity of this analytical technique in hospital facilities. This work introduces effective multinuclear and multidimensional solid-state NMR methodologies to study the complex chemical and structural properties characterizing kidney stone composition. As a basis for comparison, three hydrates ( n=1 , 2 and 3) of calcium oxalate are examined along with nine representative kidney stones. The multinuclear magic angle spinning (MAS) NMR approach adopted investigates the ${}^{1}H$ , ${}^{13}C$ , ${}^{31}P$ and ${}^{31}P$ nuclei, with the ${}^{1}H$ and ${}^{13}C$ MAS NMR data able to be readily deconvoluted into the constituent elements associated with the different oxalates and organics present. For the first time, the full interpretation of highly resolved ${}^{1}H$ NMR spectra is presented for the three hydrates, based on the structure and local dynamics. The corresponding ${}^{31}P$ MAS NMR data indicates the presence of low-level inorganic phosphate species; however, the complexity of these data make the precise identification of the phases difficult to assign. This work provides physicians, urologists and nephrologists with additional avenues of spectroscopic investigation to interrogate this complex medical dilemma that requires real, multitechnique approaches to generate effective outcomes.

## 1 Introduction

Kidney stones (KSs) are a major health problem in industrialized countries. For example, the medical costs associated with the treatment of nephrolithiasis in France exceeds EUR 800 million annually. The study of KSs is presently at the heart of a concerted multidisciplinary axis of research involving physicians, physical chemists and spectroscopists (Bazin et al., 2016). Nevertheless, the nucleation and growth of KSs remains largely unknown, and the associated mechanism is based mainly on assumption and incomplete evidence; hence, more thorough and wide-ranging structural investigations are still required (Sherer et al., 2018; Bazin et al., 2020). The growth of KSs is clearly a multifactorial problem, with their chemical composition and morphology presenting considerable variability due to the extreme complexity of the in vivo reaction media in which they are formed. The resultant biological materials exhibit very different characteristics as they can emanate from wide-ranging pathological scenarios, including bacterial infection, genetic predispositions, diabetes mellitus and bowel diseases (Bazin et al., 2012). Hence, KSs can be considered as being real examples of hybrid organic–inorganic nanocomposite materials.

The main mineral components comprising hydrated calcium oxalates are the monohydrate ${\mathrm{CaC}}_{2}O_{4}\cdot H_{2}O$ (whewellite – COM) and dihydrate ${\mathrm{CaC}}_{2}O_{4}\cdot 2H_{2}O$ (weddellite – COD) species, although amorphous calcium oxalate can also be observed (Gehl et al., 2015; Ruiz-Agudo et al., 2017). The trihydrate form, ${\mathrm{CaC}}_{2}O_{4}\cdot 3H_{2}O$ (caoxite – COT) is almost never observed in vivo but can be synthesized in an aqueous solution. COD is characterized by a zeolitic structure exhibiting a true structural challenge. It is considered as being one of the very few natural MOFs (metal organic frameworks; Huskić et al., 2016; Dazem et al., 2019), and its chemical formula is better represented by ${\mathrm{CaC}}_{2}O_{4}\cdot (2+x)H_{2}O$ ( x≤0.5 ; Petit et al.,  2018). “Structural” and “zeolitic” water molecules are, therefore, distinguished. Calcium phosphates and other mineral phases can also be detected in KSs, i.e., hydroxyapatite ( ${\mathrm{Ca}}_{10}({\mathrm{PO}}_{4}{)}_{6}(\mathrm{OH}{)}_{2}$ ), which may be partially carbonated, brushite ( ${\mathrm{CaHPO}}_{4}\cdot 2H_{2}O$ ) or struvite ( ${\mathrm{NH}}_{4}{\mathrm{MgPO}}_{4}\cdot 6H_{2}O$ ; Gardner et al., 2021). The organic components (from a few percent to a major fraction) include, e.g., proteins (collagen among them), uric acid, lipids, triglycerides, etc. The nature of the organic–inorganic interfaces remain largely unknown to date. This chemical and structural complexity at several scales requires the use of a wide variety of characterization methods. Recently, elaborate experiments took advantage of the last development in TEM (transmission electron microscopy; Gay et al., 2020) and of synchrotron radiation (Bazin et al., 2012). In hospitals, optical microscopy, Fourier transform infrared (FTIR), FTIR microscopy, SEM (scanning electron microscopy) and X-ray diffraction are used in routine mode. Curiously, solid-state NMR has been used very rarely in the context of KSs (and other pathological calcifications), apart from sparse ${}^{13}C$ and ${}^{31}P$ studies (Bak et al., 2000; Jayalakshmi et al., 2009; Reid et al., 2011, 2013; Li et al., 2016; Dessombz et al., 2016), which is unlike other human hard tissues such as bones and teeth. This is probably due to the fact that solid-state NMR instruments are not widely available in hospital settings. It is also stressed that some KSs are small so that the intrinsic lack of sensitivity associated to NMR may be a drawback. Other nuclei, such as ${}^{1}H$ and ${}^{43}\mathrm{Ca}$ , can act as potential NMR targets. However, ${}^{1}H$ solid-state NMR remains a rather specialized technique because of the relative inefficiency of magic angle spinning (MAS) in producing really high-resolution data from most systems. ${}^{43}\mathrm{Ca}$ ( I=7/2 ) is particularly insensitive (as a result of its extremely low natural abundance, ∼0.14 %, and low γ , $-1.8028\times 10^{7}$ rad s ${}^{-1}$ T ${}^{-1}$ , 57.2 MHz at 20 T).

In this work, a comprehensive multinuclear solid-state NMR approach is presented that facilitates the detailed structural analysis of KSs and the related synthetic hydrated calcium oxalate phases (COM, COD and COT) associated with their composition. The synthetic phases were obtained by carefully controlling the precipitation of calcium salts in aqueous solutions, as described below in Sect. 6 (Leroy, 2016). In total, nine KSs were studied systematically, with some of them exhibiting similar NMR fingerprints. The spectra of five of them (KS1 → KS5) are presented here. They come from the KS collection of the Tenon hospital (Paris, France) led by Michel Daudon (the collection counts tens of thousands of samples from all origins, exhibiting the largest variety of size, chemical composition and morphology worldwide). Our main goal here is to reach out to the physician community and, more specifically, nephrologists, urologists and biologists. NMR methods are presented at a moderate to high magnetic field (i.e., 7.0 to 16.4 T MHz) in order to make them much more widely accessible. Occasionally, further developments at an ultra-high magnetic field (up to 35.2 T) are proposed to the user. Particular emphasis is placed on high-resolution ${}^{1}H$ MAS NMR, with homonuclear decoupling and the complete interpretation of spectra based on structural data, and ${}^{43}\mathrm{Ca}$ MAS NMR. To the best of our knowledge, these nuclei have never been used as spectroscopic probes for KS studies (apart from a unique ${}^{43}\mathrm{Ca}$ MAS NMR study by Bowers and Kirkpatrick, 2011). A complete experimental protocol is then presented for the reconstruction of ${}^{13}C$ NMR spectra, including organic/inorganic and/or rigid/mobile components. Finally, the intriguing role of phosphates in KSs is partially deciphered by 2D ${}^{1}H{-}^{31}P$ heteronuclear correlation (HETCOR) MAS NMR experiments despite the low phosphate content in KSs.

## 2 Quick and reliable assignment of hydrated calcium oxalate and organic phases by ${}^{1}H$ high-resolution solid-state NMR experiments

### 2.1 CRAMPS (Combined Rotation And Multiple Pulses Spectroscopy) approach

In terms of NMR sensitivity, ${}^{1}H$ greatly exceeds that of ${}^{13}C$ and ${}^{43}\mathrm{Ca}$ . Moreover, it is an I=1/2 nucleus, leading to quantitative data much more rapidly if relaxation delays are carefully set. It follows that ${}^{1}H$ is a target nucleus in the study of crystalline hydrated calcium oxalate phases and KSs. Moreover, as KSs are bio-nanocomposites, ${}^{1}H$ can be considered as being a spectroscopic spy present both in the organic and inorganic components, making the study of the interfaces possible eventually. In the absence of local dynamics, the strong ${}^{1}H{-}^{1}H$ dipolar interaction is a major issue in ${}^{1}H$ solid-state NMR, leading to considerable broadening of the resonances. Current trends to reach the highest ${}^{1}H$ NMR resolution combine ultra-fast MAS, up to 111 kHz or above (Samoson, 2019), with an ultra-high magnetic field, up to 35.2 T, (Gan et al., 2017), in order to average the strong dipolar couplings. Indeed, the homogeneous character of the homonuclear dipolar interaction implies poor MAS efficiency at low to moderate spinning frequencies (Schmidt-Rohr and Spiess, 1994; note that the temperature increase, inside a 0.7 mm diameter rotor, is estimated to be roughly 20 ${}^{\circ }$ C in the fast/ultra-fast regime, i.e., $\nu_{\mathrm{rot}}>30$ kHz). This point is of prime importance as calcium oxalate structures may undergo subtle structural modifications upon heating (Deganello, 1981; Shepelenko et al., 2019; see also Sect. 6). More generally, for a 2.5 mm probe, the temperature increase is <5 ${}^{\circ }$ C at 5 kHz, and 40 ${}^{\circ }$ C at 30 kHz. For a 7 mm probe, the order of magnitude is 5 ${}^{\circ }$ C at 5 kHz.

**Figure 1:**
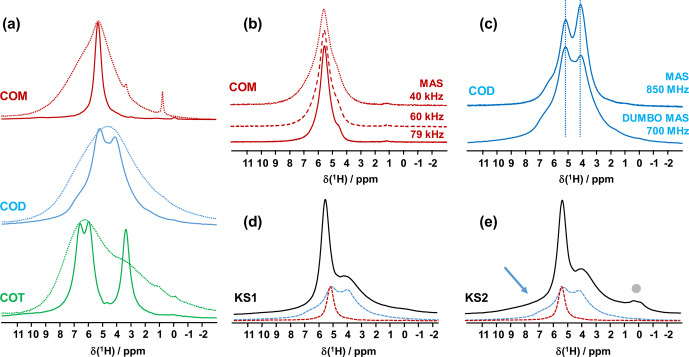
**(a)** ${}^{1}H$ MAS (dashed lines) and ${}^{1}H$ DUMBO MAS (solid lines) NMR spectra of COM (in red), COD (in blue) and COT (in green; $\nu_{\mathrm{rot}}=12$ kHz, 700 MHz, 16.4 T). Only the isotropic resonances are represented. **(b)** ${}^{1}H$ very fast MAS (40, 60 and 79 kHz) NMR spectra of COM at a very high magnetic field (850 MHz). **(c)** Comparison of the ${}^{1}H$ NMR spectra of COD obtained under DUMBO MAS ( $\nu_{\mathrm{rot}}=12$ kHz, 700 MHz) and very fast MAS ( $\nu_{\mathrm{rot}}=79$ kHz, 850 MHz) conditions. Vertical dashed lines are for illustration purposes only. **(d)** ${}^{1}H$ DUMBO MAS NMR spectrum of KS1 ( $\nu_{\mathrm{rot}}=12$ kHz, 700 MHz). The red and blue dashed lines correspond to the experimental ${}^{1}H$ DUMBO MAS NMR spectra of COM and COD, respectively. **(e)** ${}^{1}H$ DUMBO MAS NMR spectrum of KS2 ( $\nu_{\mathrm{rot}}=12$ kHz, 700 MHz). The plain light gray circle indicates the presence of organic components in KS2. The blue arrow indicates the superposition of organic components (aromatic region) and the deshielded shoulder of COD.

However, such leading edge equipment is not widely available. An alternative is to use the CRAMPS sequence at a moderate spinning frequency ( $\nu_{\mathrm{rot}}<12$ kHz; Paruzzo and Emsley, 2019). The DUMBO sequence (Decoupling Using Mind-Boggling Optimization) belongs to the CRAMPS family (Lesage et al., 2003). Using this approach, the internal temperature increase remains moderate for all rotor diameters. Moreover, this methodology can be successfully implemented on almost all magnets. Moreover, larger rotor diameters may be used, which can be interesting in terms of sensitivity. To the best of our knowledge, synthetic COM, COD and COT samples were never investigated by ${}^{1}H$ high-resolution solid-state NMR. The corresponding spectra are presented in Fig. 1. At $\nu_{\mathrm{rot}}$ = 12 kHz, standard ${}^{1}H$ MAS NMR spectra (Fig. 1a) are all characterized by very broad and almost featureless line shapes. Such spectral fingerprints are not useful for analytical purposes due to the strong overlap of the resonances. DUMBO decoupling leads to a drastic increase in resolution and to very characteristic features for each synthetic hydrate.

**Figure 2:**
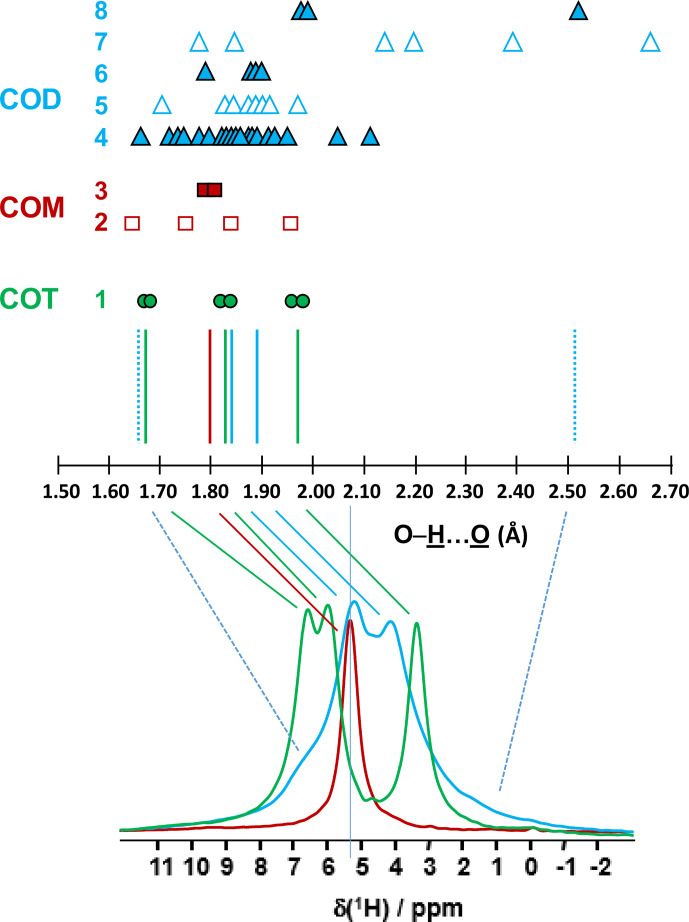
Prediction of the relative positions of $\delta_{\mathrm{iso}}({}^{1}H)$ for COM (red), COD (blue) and COT (green) as a function of the shortest $O-\underline{H}\ldots \underline{O}$ distances (in Ångström; hereafter Å). The general rules are as follows: (i) for a given $O-\underline{H}\ldots \underline{O}$ distance, a $\delta_{\mathrm{iso}}({}^{1}H)$ is associated (vertical colored solid lines), and (ii) if local dynamics are present, averaged $O-\underline{H}\ldots \underline{O}$ distances are first calculated. All distances are derived from optimized geometries at the DFT level (Table A1 and Sect. 6). The effect of eventual local dynamics in the case of the “less rigid” structure is taken into account. For line 1, the structure of COT is considered as being “rigid” (plain green circles). On the basis of the shortest $O-\underline{H}\ldots \underline{O}$ distance, the six inequivalent protons can be associated in three groups. To each group, a single average $\delta_{\mathrm{iso}}({}^{1}H)$ is assigned. A total of three lines for COT is predicted (represented by the three vertical green solid lines). For line 2, the structure of COM is considered as being less rigid (open squares), while for line 3 the corresponding averaged distances are represented by plain squares. A single average $\delta_{\mathrm{iso}}({}^{1}H)$ is associated as the averaged distances are very close. A total of one line for COM is predicted. The COD case is shown in lines 4 to 8, where COD exhibits both rigid (plain triangles; line 4) and less rigid water molecules (open triangles in line 5 for the four structural water molecules and in line 7 for the three zeolitic water molecules). For line 6, the corresponding averaged distances for the structural water molecules are represented by plain triangles, and for line 8, the corresponding averaged distances for the zeolitic water molecules are represented by plain triangles. A continuum of $\delta_{\mathrm{iso}}({}^{1}H)$ is predicted for COD. The vertical blue dashed lines correspond to the expected limits of $\delta_{\mathrm{iso}}({}^{1}H)$ . The two vertical solid blue lines correspond to local maxima, adding lines 4, 6 and 8. At the bottom, the superposition of the ${}^{1}H$ DUMBO MAS NMR spectra for COM (red), COD (blue) and COT (green; see Fig. 1a) is shown. The solid and dashed lines connect the experimental data and the predicted $\delta_{\mathrm{iso}}({}^{1}H)$ .

**Figure 3:**
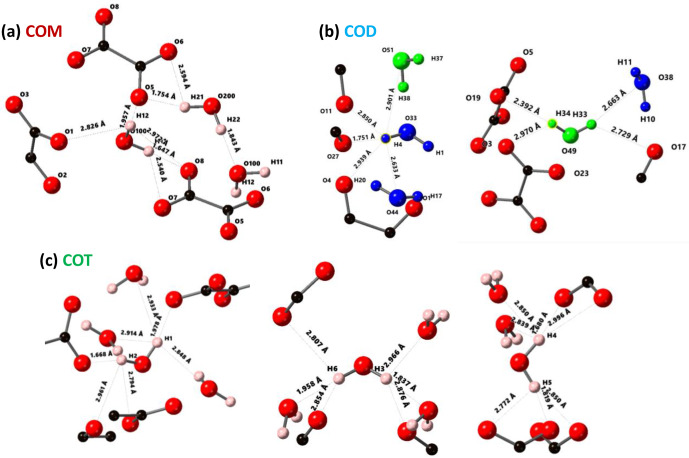
Structural details of COM **(a)**, COD **(b)** and COT **(c)**. For each proton of the water molecules, the shortest $O-\underline{H}\ldots \underline{O}$ distance (in Å) is represented, together with the number of $\underline{H}\ldots \underline{O}$ contacts (3 Å cut-off). For COT, the number of $\underline{H}\ldots \underline{O}$ contacts is high (three to four), and the COT structure is considered as being rigid. In the case of COD, the structural and zeolitic water molecules are represented in blue and green, respectively. A selection of rigid (H4) and less rigid (H33 or H34) water molecules is presented. All distances and the number of $\underline{H}\ldots \underline{O}$ contacts are summarized in Table A1. Color code: red is O, black is C and light pink is H.

The COT crystallographic structure exhibits six inequivalent sites for protons (Heijnen et al., 1985), whereas only three resonances are clearly observed at $\delta_{\mathrm{iso}}({}^{1}H)=3.36$ , 5.95 and 6.53 ppm (parts per million; Fig. 1a). A realistic assumption is that some resonances are so close that they cannot be distinguished even under DUMBO decoupling. It has been shown previously (Eckert et al., 1988; Pourpoint et al., 2007) that $\delta_{\mathrm{iso}}({}^{1}H)$ can be related to the shortest $O-{\underline{H}}^{\ldots }\underline{O}$ bond length in hydrogen bond networks. The general trend is that $\delta_{\mathrm{iso}}({}^{1}H)$ strongly increases with the shortening of $O-\underline{H}\ldots \underline{O}$ . Interestingly, the six nonequivalent hydrogens can be distinguished based on $O-{\underline{H}}^{\ldots }\underline{O}$ distances, leading to three distinct groups (Fig. 2 and Table A1 in the Appendix), namely 1.668–1.679 Å/1.809–1.837 Å/1.957–1.978 Å. It is stressed here that the distances were obtained after extensive optimization of the geometry of the COT structure at density functional theory (DFT) level (the same comment holds for the COM and COD structures; see Sect. 6). According to the literature (Pourpoint et al., 2007), a variation in $O-{\underline{H}}^{\ldots }\underline{O}$ of ∼0.3 Å is related to a $\delta_{\mathrm{iso}}({}^{1}H)$ variation of ∼3.5 ppm, which is in rather good agreement with the results presented here (i.e., the shorter the distance, the higher the isotropic ${}^{1}H$ chemical shift). We mention also (Table A1) that each proton of the structure is involved in a relatively high number of ${\underline{H}}^{\ldots }\underline{O}$ contacts (from three to four, with $O-{\underline{H}}^{\ldots }\underline{O}\leq 3$ Å). The 3Å cut-off is realistic when considering “weak” H bonds (Steiner, 2002). In other words, the shortest $O-{\underline{H}}^{\ldots }\underline{O}$ distance directly dictates $\delta_{\mathrm{iso}}({}^{1}H)$ , whereas the number of ${\underline{H}}^{\ldots }\underline{O}$ contacts is more representative of the electrostatic/dispersion contributions at a given H position (Steiner, 2002). As all protons of COT are characterized by a large number of ${\underline{H}}^{\ldots }\underline{O}$ contacts, we assume a certain character of “rigidity” in the structure at room temperature and very limited local dynamics (Fig. 3c). Under this simple assumption, at most three resolved $\delta_{\mathrm{iso}}({}^{1}H)$ are expected due to similarities in $O-{\underline{H}}^{\ldots }\underline{O}$ distances (see above), which is in good agreement with the experimental data (Fig. 1a). Therefore, the ${}^{1}H$ COT assignments are the following, using the numbering given in Table A1: 3.36 ppm for H1/H6, 5.95 ppm for H3/H5 and 6.53 ppm for H2/H4. We note that partial deuteration could be of great help in increasing the resolution further.

In the case of COM, the four H crystallographic sites (H11, H12, H21 and H22; Deganello, 1981) are characterized by a large range of $O-\underline{H}\ldots \underline{O}$ distances (from 1.647 to 1.957 Å) and a restricted number of ${\underline{H}}^{\ldots }\underline{O}$ contacts (from 1 to 3; Table A1). Therefore, a less rigid structure is expected at room temperature (when compared to COT). Rapid flips of $H_{2}O$ molecules could lead to partial averaging of $\delta_{\mathrm{iso}}({}^{1}H)$ of protons belonging to the same molecule (Fig. 3). Using the numbering given in Table A1, the average $O-{\underline{H}}^{\ldots }\underline{O}$ distances for H11/H12 and H21/H22 are very similar (i.e., 1.802 and 1.795 Å, respectively). A unique resonance is therefore expected, which is in full agreement with the ${}^{1}H$ DUMBO MAS NMR spectrum of COM (one resonance centered at 5.26 ppm; Figs. 1a and 2). Data obtained at 100 K (see Fig. A2) demonstrated the presence of four resolved ${}^{1}H$ resonances for COM. It is worth noting that $\delta_{\mathrm{iso}}({}^{1}H)$ for H11, H12, H21 and H22 in COM and H3/H5 in COT are experimentally in very close agreement with the associated $O-{\underline{H}}^{\ldots }\underline{O}$ distances. From one synthetic sample to the other, $\delta_{\mathrm{iso}}({}^{1}H)$ may vary slightly under DUMBO conditions ( ∼0.3 ppm). COM is always obtained as a final product, as shown by the X-ray powder diffraction (XRD). Subtle variations are observed, depending on the degree of the disorder present, as demonstrated very recently by Shepelenko et al. (2019).

The ${}^{1}H$ spectrum of COD (Fig. 1a) is a priori complex, as it corresponds to the superposition of structural and zeolitic water molecules (Tazzoli and Domeneghetti, 1980; Izatulina et al., 2014). It is much broader than the spectra corresponding to COM and COT. More specific features centered at $\delta_{\mathrm{iso}}({}^{1}H)=4.11$ , 5.17 and ∼6.5 (shoulder) ppm are observed (Fig. 1a). When compared to the COT spectrum, the spectral resolution decreases, as expected, from the partial disorder of the zeolitic water molecules. The detailed examination of selected $O-{\underline{H}}^{\ldots }\underline{O}$ distances (Table A1) allowed us to propose a partial assignment of the resonances. For that purpose, a model (relaxed at the DFT level), which corresponds to ${\mathrm{CaC}}_{2}O_{4}\cdot (2+0.375)H_{2}O$ , was first calculated (or ${\mathrm{Ca}}_{8}C_{16}O_{32}(H_{2}O{)}_{16}$ ( ${\text{H}}_{2}\text{O}$ ) ${}_{3}$ ). The water molecules located in the channels of the zeolitic structure are represented in italics in the preceding formula. Taking into account the number of ${\underline{H}}^{\ldots }\underline{O}$ contacts (in full analogy with the approach described above for COM and COT), among the 19 water molecules, seven molecules are considered as being less rigid (or potentially mobile), of which four of them are structural and three are zeolitic. The remaining 12 water molecules are considered as being rigid. Typical example of rigid (H4) and less rigid (H33/H34) water molecules are presented in Fig. 3b. From Fig. 2, it is then possible to predict the expected ranges of $\delta_{\mathrm{iso}}({}^{1}H)$ for COD. The rules applied are that rigid water molecules correspond to two distinct $\delta_{\mathrm{iso}}({}^{1}H)$ (line 4), whereas less rigid water molecules correspond to a single average $\delta_{\mathrm{iso}}({}^{1}H)$ (averaging of line 5 gives line 6; averaging of line 7 gives line 8). The sum of lines 4, 6 and 8 (in blue) corresponds to the expected ${}^{1}H$ spectrum for COD. On this basis, it is expected (i) that $\delta_{\mathrm{iso}}({}^{1}H)$ is distributed over a much larger range when compared to COM and COT and (ii) that some maxima should be observed (at least two, corresponding to large number of overlapping triangles in Fig. 2). Points (i) and (ii) are in very good agreement with the experimental observations. All in all, the relative predicted positions for $\delta_{\mathrm{iso}}({}^{1}H)$ resonances are in agreement with the experimental data for COM, COD and COT (bottom of Fig. 2), validating the proposed assignments.

As a first conclusion of this section, ${}^{1}H$ DUMBO MAS NMR spectra for COM, COD and COT correspond to useful fingerprints for analytical purposes as they are clearly characteristic for each phase. Such fingerprints can be used for the analysis of ${}^{1}H$ NMR spectra of KSs (see below). We emphasize that ${}^{1}H$ spectra with an excellent signal-to-noise ratio were obtained within minutes. As $\delta_{\mathrm{iso}}({}^{1}H)$ values are very sensitive to H-bond networks and to local motional averaging, studies performed on synthetic COM, COD and COT were necessary prior to the detailed analyses of KSs. Nevertheless, the following comments have to be made at this stage: (i) first, the ${}^{1}H$ DUMBO MAS methodology ( $\nu_{\mathrm{rot}}=12$ kHz, 700 MHz) is comparable to the very fast MAS/very high magnetic field approach ( $\nu_{\mathrm{rot}}∼80$ kHz, 850 MHz) without any multiple pulses decoupling. In Fig. 1b, the ${}^{1}H$ MAS NMR spectra of COM are presented at various fast/very fast rotation frequencies, from $\nu_{\mathrm{rot}}=40$ to ∼80 kHz. As in the case of the DUMBO MAS approach, a single resonance (with a small shoulder) was observed, showing a continuously decreasing linewidth with increasing the MAS frequency. The linewidth obtained at ∼80 kHz is still broader than the one observed under DUMBO MAS conditions. In Fig. 1c, the two approaches are compared in the case of COD. The resolution is slightly enhanced under very fast MAS at 79 kHz, but it remains comparable to DUMBO conditions at 12 kHz. More importantly, the relative intensities are not strictly preserved, indicating that some distortions of the line shapes may occur under DUMBO conditions. It follows that only semi-quantitative data can be extracted, at best, in the case of complex mixtures of hydrated calcium oxalate phases. Moreover, dynamics at room temperature may impact the efficiency of the DUMBO decoupling.

Finally, two KSs (KS1 and KS2) were studied by ${}^{1}H$ DUMBO MAS NMR (Fig. 1d and e). In the case of KS1, a mixture of COM and COD is immediately detected (in full agreement with FTIR and XRD; not shown here). As stated above, a slight deviation of $\delta_{\mathrm{iso}}({}^{1}H)$ for COM is observed. A semi-quantitative analysis of the COM/COD proportions is possible and could be systematically compared to FTIR analyses (as routinely obtained in hospitals). In addition to COM and COD resonances, the ${}^{1}H$ DUMBO MAS NMR spectrum of KS2 exhibits small new contributions that can be attributed to organic moieties (such as proteins). In this case, a semi-quantitative analysis of the KSs appears more difficult to perform. Working at much higher magnetic field, 35.2 T (Gan et al., 2017), and under ultra-fast MAS ( ≫100 kHz) should lead to increased resolution and easier direct quantification of the spectra. Finally, we mention the fact that DUMBO experiments may be sensitive to local dynamics, especially in the intermediate regime (Paruzzo and Emsley, 2019). It may involve some discrepancies between ${}^{1}H$ and ${}^{13}C$ NMR data in terms of quantification (see Sect. 4).

### 2.2 $T_{2}^{*}({}^{1}H)$ editing and ${}^{1}H{-}^{1}H$ double-quantum (DQ) experiments

**Figure 4:**
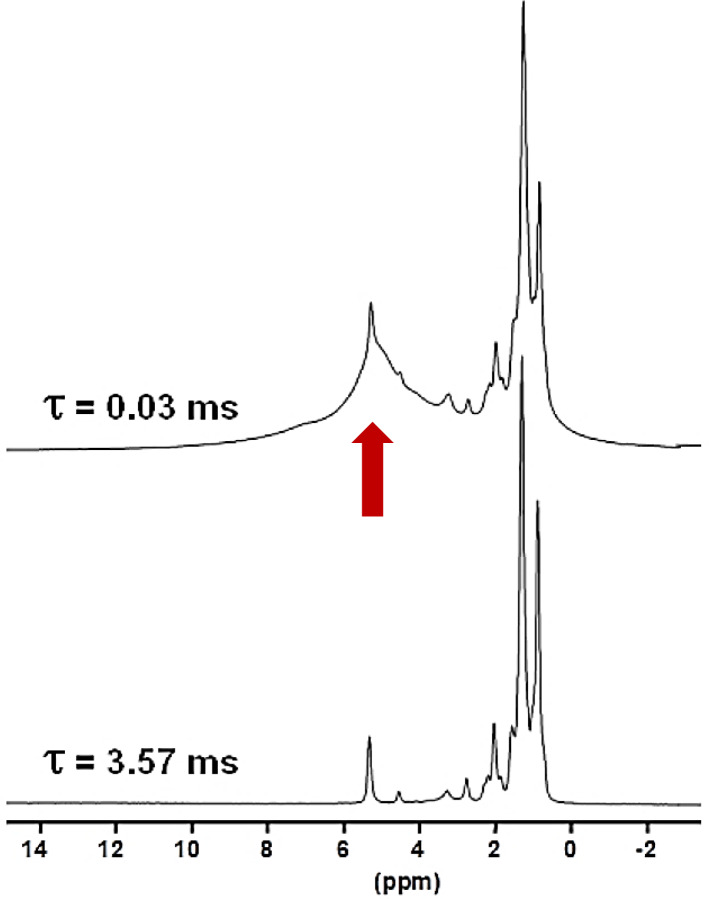
${}^{1}H$ Hahn echo MAS NMR spectra for KS3 recorded at 16.4 T. τ was synchronized with the rotation frequency, which is shown here as $\nu_{\mathrm{rot}}=30$ kHz. No temperature control was implemented, leading to a ∼40 ${}^{\circ }$ C increase in the sample temperature and the associated increase in local dynamics. The vertical red arrow corresponds to the resonance coming from COM (see also Fig. 1a and b).

Another KSs sample, KS3, that was studied contained a large proportion of organic moieties (as shown by FTIR). Another option to increase the ${}^{1}H$ NMR resolution is to implement standard Hahn echoes with increasing delays, τ , (up to several milliseconds) and synchronization with the MAS frequency. The magnetization associated to the protons characterized by short $T_{2}^{*}({}^{1}H)$ will dephase very rapidly. ${}^{1}H$ MAS echoes ( $\nu_{\mathrm{rot}}=30$ kHz) for KS3 are presented in Fig. 4. From XRD data (not shown here) confirmed by ${}^{13}C$ CP MAS NMR data (see Sect. 4), KS3 contains COM as a major mineral phase. For long τ , sharp lines (associated to mobile components) were obtained, whereas the broader COM component, around 5.2 ppm, was totally suppressed. $\delta_{\mathrm{iso}}({}^{1}H)$ values agree with unsaturated fatty acids ( $\delta_{\mathrm{iso}}({}^{1}H)∼5.25$ ppm; Ren et al., 2008). The presence of triglycerides is excluded as the $C\underline{H}$ and $C{\underline{H}}_{2}$ resonances of the glycerol backbone ( $\delta_{\mathrm{iso}}({}^{1}H)∼5.0$ and 4.0 ppm, respectively) were not detected.

**Figure 5:**
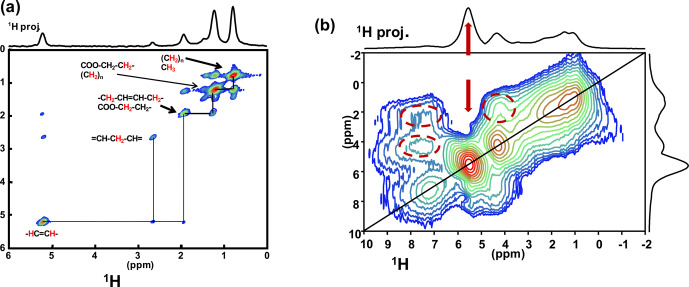
**(a)** ${}^{1}H{-}^{1}H$ DQF COSY MAS NMR spectrum for KS3 at $\nu_{\mathrm{rot}}=30$ kHz recorded at 16.4 T. Here, no temperature control was implemented, leading to a ∼40 ${}^{\circ }$ C increase in the local temperature and, therefore, of local dynamics. All peaks are assigned to contributions from unsaturated mobile fatty acids (with unsaturations). **(b)** ${}^{1}H{-}^{1}H$ DQ BABA MAS NMR spectrum for KS3 at $\nu_{\mathrm{rot}}=79$ kHz recorded at 16.4 T (no temperature control; SQ–SQ, single quantum–single quantum, representation). The recoupling period is two rotor periods. Off-diagonal correlations (immobile organic moieties) are highlighted by dashed red ovals. The red arrows indicate the COM contribution.

Such a level of resolution allowed for the implementation of J-MAS-derived pulse schemes, such as the ${}^{1}H{-}^{1}H$ double quantum-filtered (DQF) correlation spectroscopy (COSY) MAS experiment (based on isotropic $J{(}^{1}H{-}^{1}H)$ couplings). This experiment is part of the toolbox for a more general dynamics-based spectral editing research topic applied to biological solids (Mroue et al., 2016; Matlahov and van der Wel, 2018; Gopinath and Veglia, 2018). The ${}^{1}H{-}^{1}H$ DQF COSY MAS spectrum is presented in Fig. 5a for KS3. All resonances of the mobile fatty acid chains were assigned in a straightforward way, demonstrating the pertinence of this through-bond correlation experiment. On the other hand, dipolar-based double quantum (DQ) experiments can be implemented to establish through-space proximities between protons (such as back to back or BABA; Feike et al., 1996). It is a distinct advantage to perform such experiments under very fast MAS (here 79 kHz). Indeed, the spectral resolution is drastically increased, leading to an easier observation of the correlation peaks. The ${}^{1}H{-}^{1}H$ DQ BABA MAS NMR spectrum of KS3 is presented in Fig. 5b. The ${}^{1}H$ resonance corresponding to the COM phase is clearly evidenced on the ${}^{1}H$ projection and on the 2D diagonal (red arrows). Moreover, red dashed ovals indicate correlations involving the protons of the immobile proteins contained in KS3 (essentially the ${{}^{1}H}^{N}{-}^{1}H^{\alpha }$ , ${}^{1}H^{\alpha }{-}^{1}H^{\beta }$ regions). ${}^{1}H$ spin diffusion experiments should help to highlight actual correlations between the organic and inorganic components at the interface (Schmidt-Rohr and Spiess, 1994).

## 3 Natural abundance ${}^{43}\mathrm{Ca}$ solid-state NMR experiments

Natural abundance solid-state ${}^{43}\mathrm{Ca}$ MAS NMR spectroscopy remains a challenge. Indeed, the NMR characteristics of this quadrupolar nucleus ( I=7/2 ) are clearly unfavorable, as natural abundance is 0.14 % and low γ ( $\nu_{0}=57.2$ MHz at 20 T). Nevertheless, the following four main experimental approaches have been successfully developed during the last few years: (i) using large volume rotors (7 mm; ∼400 mg of sample) at high magnetic field (20 T), under moderate MAS ( ∼5 kHz) and implementing DFS (double frequency sweep) excitation scheme (Sect. 6); (ii) using much smaller rotors (3.2 mm; ∼20 mg of sample at ultra-high magnetic field (35.2 T) and under moderate/fast MAS ( ∼18 kHz); (iii) using dynamic nuclear polarization (DNP) to strongly enhance the ${}^{43}\mathrm{Ca}$ polarization, usually in the indirect mode (from ${}^{1}H$ to ${}^{43}\mathrm{Ca}$ ); (iv) using labeling in ${}^{43}\mathrm{Ca}$ (starting from an enriched calcite precursor; Laurencin et al., 2021; Smith, 2020; Laurencin and Smith, 2013). Here, we follow the approach in (i), which is by far the easiest to implement in most NMR facilities worldwide (as long as a low γ probe is available).

**Figure 6:**
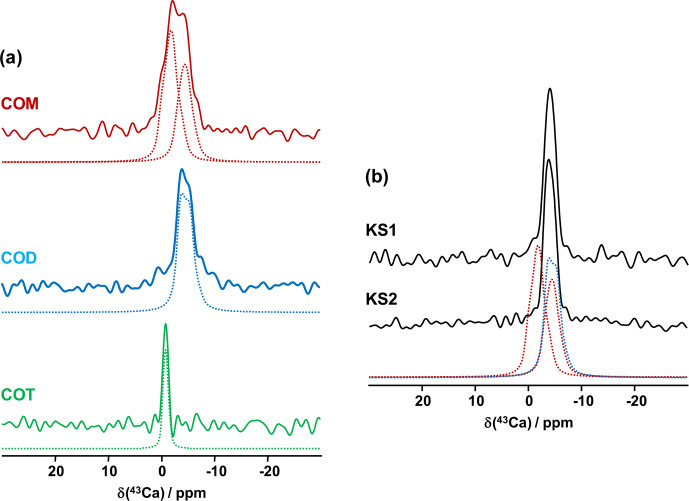
**(a)** Natural abundance ${}^{43}\mathrm{Ca}$ MAS NMR spectra of COM (red), COD (blue) and COT (green) recorded at 20.0 T ( $\nu_{\mathrm{rot}}=3$ to 5 kHz). The dashed lines correspond to fits. **(b)** Natural abundance ${}^{43}\mathrm{Ca}$ MAS NMR spectra of KS1 and KS2. The red dashed lines correspond to the two resonances associated to COM. The blue dashed line corresponds to the ${}^{43}\mathrm{Ca}$ MAS NMR spectrum of COD.

The first contributions related to the study of synthetic calcium oxalates hydrates by ${}^{43}\mathrm{Ca}$ MAS NMR spectroscopy were proposed by Wong et al. (2006) for COT and by Bowers and Kirkpatrick (2011) for the three hydrated phases. The latter claimed that the COM line shape could be attributed to an averaged Gaussian signal due to a local disorder in the structure (Tazzoli and Domeneghetti, 1980). Colas et al. (2013) demonstrated that a high signal-to-noise ratio is necessary to extract reliable quadrupolar parameters from natural abundance ${}^{43}\mathrm{Ca}$ MAS NMR spectra and reinvestigated the COM phase. Instead of a Gaussian contribution, two distinct resonances were evidenced, which is in agreement with the crystallographic data ( $\delta_{\mathrm{iso}}({}^{43}\mathrm{Ca})=-2.6$ ppm, $C_{Q}=1.50$ MHz, $\eta_{Q}=0.60$ ; $\delta_{\mathrm{iso}}({}^{43}\mathrm{Ca})=0.7$ ppm, $C_{Q}=1.60$ MHz, $\eta_{Q}=0.70$ ). The ${}^{43}\mathrm{Ca}$ MAS NMR spectra of COM, COD and COT, recorded at 20.0 T, are presented in Fig. 6a. All spectra were obtained in natural abundance in a reasonable amount of experimental time ( ∼2 h for COM and COT; ∼4 h for COD). The ${}^{43}\mathrm{Ca}$ NMR fingerprints obtained allow for unambiguous distinctions of the three phases. The sharpest line (characterized by the smallest C ${}_{Q}$ ) is observed for COT (one unique crystallographic site). For this particular phase, second-order quadrupolar broadening is efficiently suppressed at 20 T, leading directly to $\delta_{\mathrm{iso}}({}^{43}\mathrm{Ca})=-0.1$ ppm. This value is slightly different from the one reported by Wong et al. (2006; i.e., -4.2 ppm). Such a discrepancy can be attributed to a difference in chemical shift referencing (Gervais et al., 2008). The associated quadrupolar parameters for COT (Wong et al., 2006) were $C_{Q}=1.55$ MHz, $\eta_{Q}=0.72$ . $C_{Q}$ is probably overestimated, as such a value would definitely produce second-order quadrupolar broadening under MAS at 20.0 T (see above the quadrupolar parameters for COM). Finally, a rather featureless spectrum is obtained for COD (one crystallographic site), exhibiting a much larger linewidth than for COT ( $\delta_{\mathrm{iso}}({}^{43}\mathrm{Ca})∼-2.6$ ppm, $C_{Q}∼1.60$ MHz, $\eta_{Q}∼0.20$ ). We assign this broadening to the distribution of zeolitic water molecules (leading consequently to a slight distribution of $\delta_{\mathrm{iso}}({}^{43}\mathrm{Ca}))$ . Hence, it is demonstrated that natural abundance ${}^{43}\mathrm{Ca}$ MAS NMR spectroscopy is useful for characterizing hydrated calcium oxalate phases. The use of moderate MAS is sufficient to retrieve a satisfactory resolution as characteristic $C_{Q}({}^{43}\mathrm{Ca})$ are usually small/very small ( <1.8 MHz). However, the $\delta_{\mathrm{iso}}({}^{43}\mathrm{Ca})$ range covered by these three phases is making this NMR parameter less sensitive to distinguishing the hydrates. This comes from the fact that $\delta_{\mathrm{iso}}({}^{43}\mathrm{Ca})$ is mainly determined by the coordination of the Ca atoms and the mean <Ca-O> distances. These parameters are almost identical for COM, COD and COT (eight-fold coordination for COM and COD; seven-fold coordination for COT; range of averaged Ca-O distances, i.e., 2.47–2.49 Å). This last comment is rather in contradiction with previous conclusions proposed in the literature (Bowers and Kirkpatrick, 2011).

**Figure 7:**
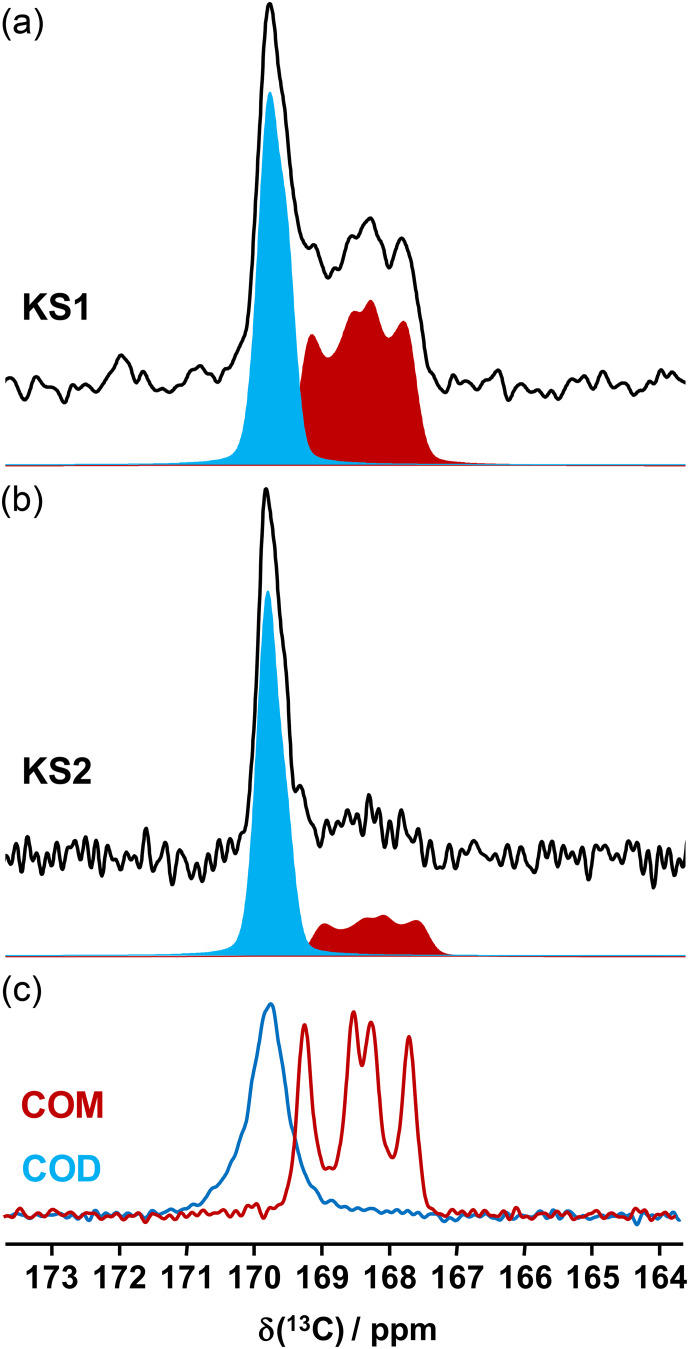
${}^{13}C$ CP MAS NMR spectra of **(a)** KS1 and **(b)** KS2 (recorded at 16.4 T, $\nu_{\mathrm{rot}}=5$ kHz) and the corresponding COM (red) and COD (blue) contributions. **(c)** ${}^{13}C$ CP MAS NMR spectra of the synthetic COM (red line) and COD (blue line) recorded under similar conditions.

The natural abundance ${}^{43}\mathrm{Ca}$ MAS NMR spectra of KS1 and KS2 are presented in Fig. 6b. They are largely similar to the COD spectrum overall. The contribution of a COM component is hardly discernable (though it is present, especially in KS1; see Fig. 7). As stated above, the structure of COM is subject to subtle structural variations which could lead to the overlap of the two ${}^{43}\mathrm{Ca}$ resonances. In other words, though interesting in principle, natural abundance ${}^{43}\mathrm{Ca}$ MAS NMR spectroscopy (inherently associated with the limited signal-to-noise ratio) should not be used as a first solid-state NMR tool of investigation for KSs. However, ${}^{43}\mathrm{Ca}$ NMR would benefit from working at a ultra-high magnetic field (35 T) in order to drastically increase the resolution and enhance ${}^{43}\mathrm{Ca}$ NMR analytic capabilities.

## 4 Back to ${}^{13}C$ NMR: spectral edition and reconstruction of spectra

${}^{13}C$ NMR data related to synthetic calcium oxalate phases and KSs are the most represented in the literature. This is probably due to the fact that the spectral resolution is high under MAS and that CP (cross-polarization) MAS experiments can easily be implemented even at low or moderate magnetic field. Typical ${}^{13}C$ CP MAS NMR spectra for COM and COD are presented in Fig. 7. A total of four isotropic resonances are observed for COM, as expected from XRD data (Colas et al., 2013), and one unique broader resonance is observed for COD, as expected from XRD data, considering the disorder associated to the zeolitic water molecules. Such a disorder has an impact on the resolution of the ${}^{13}C$ NMR spectra.

It is observed that the chemical shift range of interest is very restricted ( ∼4 ppm from 167 to 171 ppm), corresponding to ∼0.8 % of the whole ${}^{13}C$ isotropic chemical shift range. ${}^{13}C$ CP MAS NMR spectra for KS1 and KS2 are also presented in Fig. 7. The presence of COM and COD components is clearly evidenced and could be quantified if necessary (by increasing the signal-to-noise ratio, S/N , significantly). The S/N is adequate here for coarse quantification. For better accuracy, a longer experimental time will be necessary. Moreover, NMR experiments will be combined with denoising techniques developed recently by Laurent and Bonhomme (2020).

**Figure 8:**
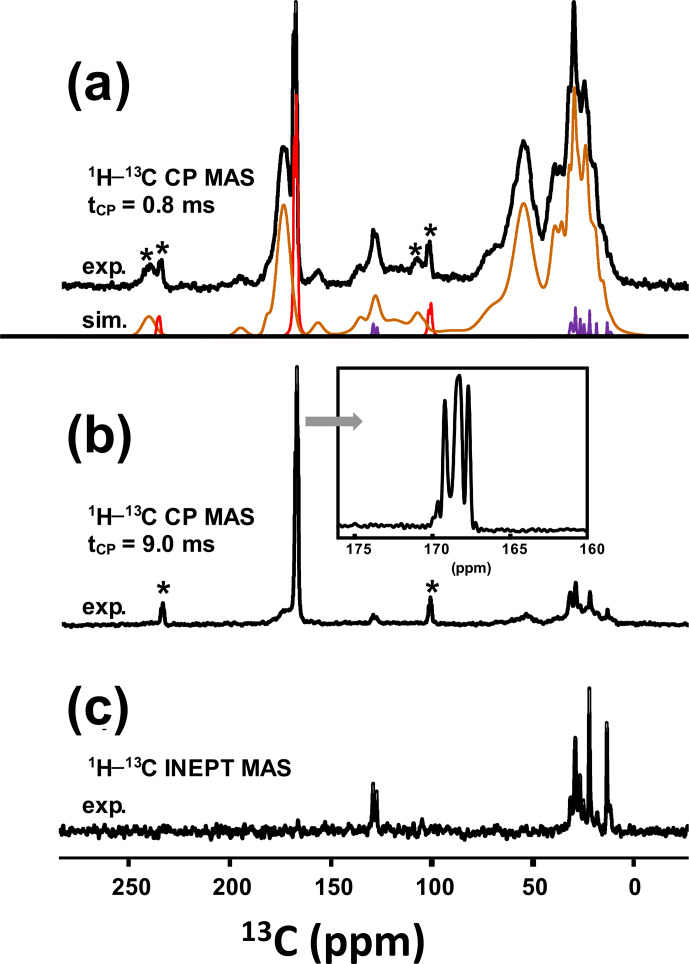
**(a)** ${}^{13}C$ CP MAS NMR spectrum of KS3 (recorded at 7.0 T using a short contact time, i.e., 0.8 ms; $\nu_{\mathrm{rot}}=5$ kHz). The experimental spectrum is decomposed into the following three components: COM (in red), fatty acids (in purple) and proteins (in brown). **(b)** ${}^{13}C$ CP MAS NMR spectrum of KS3 (recorded at 7.0 T using a long contact time, i.e., 9.0 ms). The inset highlights the COM contribution (four resonances, with two of them almost overlapping; Colas et al., 2013). **(c)** ${}^{1}H{-}^{13}C$ refocused INEPT J-MAS NMR spectrum of KS3 (recorded at 7.0 T). The unsaturations of the fatty acids are clearly evidenced at $\delta_{\mathrm{iso}}({}^{13}C)∼130$ ppm. The asterisk ( ${}^{*}$ ) indicates the spinning sidebands.

As a matter of fact, a single experiment at fixed contact time (usually >5 ms) is sufficient, in principle, for quantitative purposes, as ${}^{1}H{-}^{13}C$ dipolar couplings are comparable for all ${}^{13}C$ sites (differences in relative intensities can be evidenced at much short contact time, i.e., <0.5 ms). The case of KS3 is by far more complex. As stated in Sect. 1, a given KS may include a complex organic component containing lipids, triglycerides, membrane components, glycoproteins (like the Tamm–Horsfall protein) and glycosaminoglycans, among other species (Reid et al., 2011). The approximate chemical composition of KS3 is ∼10 % proteins, ∼20 %–25 % COM and ∼65 % amorphous silica (Dessombz et al., 2016). In Fig. 8, we propose a robust protocol to reconstruct the ${}^{13}C$ MAS NMR spectra starting from well-identified subspectra. At a short contact time (0.8 ms), all carbon-containing species are detected, corresponding to both sharp and broad lines (Fig. 8a).

Then, a $T_{1\rho }({}^{1}H)$ filter was applied by increasing the contact time by a factor of ∼10 , leading to the drastic reduction in the intensities of the broad components. The four resonances of COM are clearly observed (insert in Fig. 8b). COD is absent, which is in agreement with powder XRD and FTIR data. It follows that the proton spin baths corresponding to COM and the broad components are independent; spin diffusion and domain size measurements could be implemented as complementary experiments (Schmidt-Rohr and Spiess, 1994). The 1D ${}^{1}H{-}^{13}C$ refocused INEPT J-MAS NMR sequence (Fig. 8c) allowed a selective extraction of the mobile components corresponding to the fatty acids (see also Fig. 5a). The unsaturated nature is clearly evidenced by the shift at $\delta_{\mathrm{iso}}({}^{13}C)∼130$ ppm. Finally, the ${}^{13}C$ CP MAS NMR spectrum (Fig. 8a; bottom) could be reconstructed with resonances from (i) the COM phase and its associated spinning sidebands (in red), (ii) fatty acids characterized by very sharp lines (in purple) (iii) and proteins (in brown; Cavanagh et al., 2007), for which a precise attribution cannot be given at this stage.

## 5 The ubiquitous (but elusive) presence of phosphorus in KSs: ${}^{31}P$ MAS and CP MAS experiments

**Figure 9:**
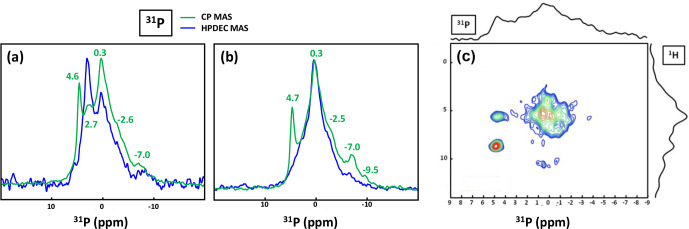
**(a)** ${}^{31}P$ MAS under high-power $\left\{{}^{1}H\right\}$ decoupling (in blue) and CP MAS (in green) under high-power $\left\{{}^{1}H\right\}$ decoupling NMR spectra of KS4. Some specific chemical shifts are highlighted. **(b)** ${}^{31}P$ MAS under high-power $\left\{{}^{1}H\right\}$ decoupling (in blue) and CP MAS (in green) NMR spectra of KS5 (representative of an ensemble of five KSs). Some specific chemical shifts are highlighted. **(c)** ${}^{1}H{-}^{31}P$ HETCOR CP MAS NMR spectrum of KS4 (temperature control at -20 ${}^{\circ }$ C). All spectra shown here were recorded at 16.4 T.

**Figure A1:**
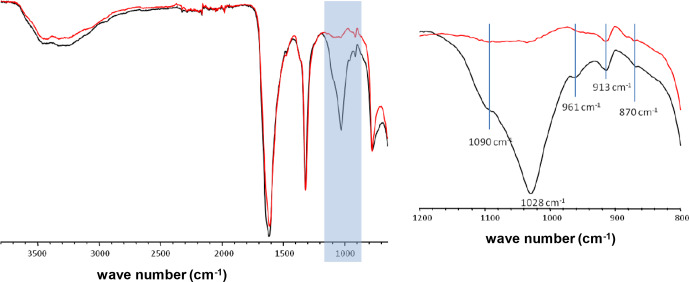
FTIR spectra of two KSs containing a mixture of COM and COD phases. The main difference lies in the light blue wave number region corresponding to phosphate vibrations (913, 961, 1090 cm ${}^{-1}$ hydroxyapatite; 870 cm ${}^{-1}$ carbonates).

**Figure A2:**
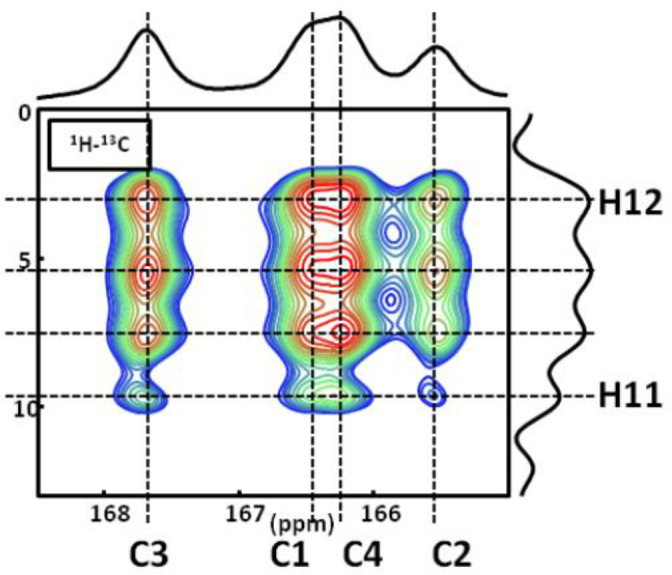
${}^{1}H{-}^{13}C$ CP DNP HETCOR MAS NMR spectrum of COM at T=100 K. In total, four distinct ${}^{1}H$ resonances are clearly evidenced on the ${}^{1}H$ indirect dimension. The contact time is 9.0 ms, and 16 ${}^{1}H{/}^{13}C$ correlations are observed. Note: the temperature used (100 K) has an impact on the values of the ${}^{13}C$ chemical shifts. The main goal here is to demonstrate that four ${}^{1}H$ resonances are clearly observed in the indirect dimension.

Bak et al. (2000) used ${}^{31}P$ MAS and CP MAS experiments to evidence phosphate-containing phases in KSs. The presence of phosphate groups in KSs is not unusual and is observed mainly by FTIR (Fig. A1). However, their exact chemical nature remains unclear. Phosphates in KSs can correspond to (i) mineral phases, such as substituted (carbonated) hydroxyapatite ( ${\mathrm{Ca}}_{10}({\mathrm{PO}}_{4}{)}_{6}(\mathrm{OH}{)}_{2}$ ), brushite ( ${\mathrm{CaHPO}}_{4}\cdot 2H_{2}O$ ) or struvite ( ${\mathrm{NH}}_{4}{\mathrm{MgPO}}_{4}\cdot 6H_{2}O$ ), and (ii) organic phosphates present in phospholipids (in the cell membrane) and/or DNA, RNA and adenosine triphosphate (ATP) molecules (Butusov and Jernelöv, 2013). Usually, phosphates are found as minor components in KSs, making ${}^{31}P$ NMR attractive given the high inherent signal sensitivity of ${}^{31}P$ (which is also an I=1/2 nucleus). A total of six KSs (exhibiting COM as the major phase and the apparent absence of phosphate phases by powder XRD) were studied here. The representative ${}^{31}P$ MAS and CP MAS NMR spectra of the KSs are presented in Fig. 9.

**Figure A3:**
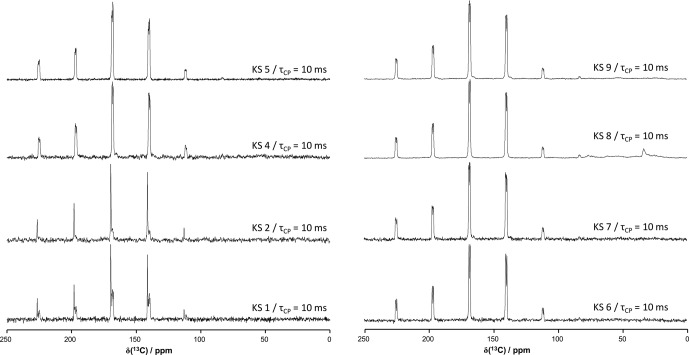
${}^{13}C$ CP MAS NMR spectra of all KSs presented in this work (700 MHz). The contact time in milliseconds is indicated systematically.

**Figure A4:**
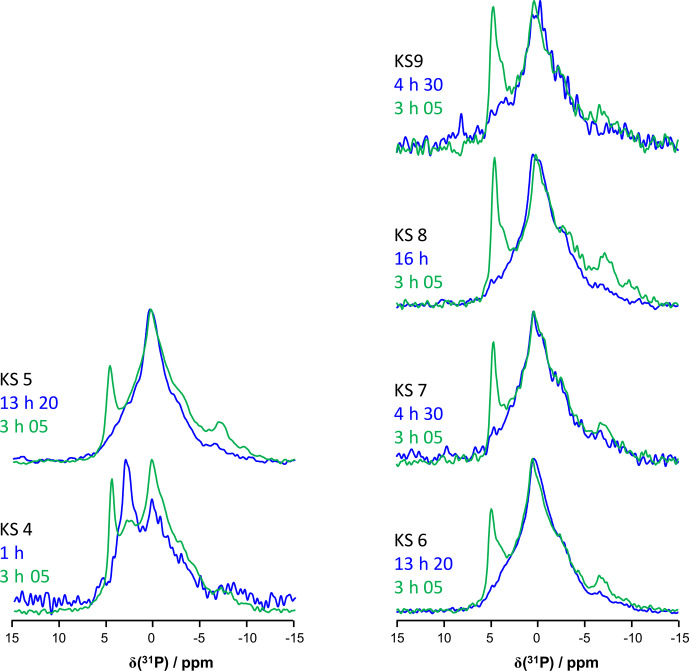
${}^{31}P$ MAS and CP MAS NMR spectra of all KSs presented in this work (700 MHz).

The ${}^{31}P$ NMR fingerprint of KS4 is specific (Fig. 9a), whereas KS5 has a ${}^{31}P$ fingerprint analogous to four other KSs (Fig. 9b). The acquisition time is ∼2 to 3 h, demonstrating that the amount of phosphate species is indeed small in all samples. One notes a large distribution of $\delta_{\mathrm{iso}}({}^{31}P)$ , corresponding not only to structural disorder but also to strong chemical variability. In order to facilitate the assignment of $\delta_{\mathrm{iso}}({}^{31}P)$ , ${}^{1}H{-}^{31}P$ HETCOR CP MAS NMR experiments under active temperature control ( T=-20 ${}^{\circ }$ C) were implemented as well (Fig. 9c). In total, three clear correlations were observed, i.e., $\delta_{\mathrm{iso}}({}^{31}P)=4.6\;\mathrm{ppm}↔\delta_{\mathrm{iso}}({}^{1}H)=8.7\;\mathrm{ppm}$ , $\delta_{\mathrm{iso}}({}^{31}P)=4.6\;\mathrm{ppm}↔\delta_{\mathrm{iso}}({}^{1}H)=5.7\;\mathrm{ppm}$ and $\delta_{\mathrm{iso}}({}^{31}P)∼0.25$ – $0.30\;\mathrm{ppm}↔\delta_{\mathrm{iso}}({}^{1}H)∼5.0\;\mathrm{ppm}$ . Reasonable assignments are the following (Godinot et al., 2016): (i) the peak centered at $\delta_{\mathrm{iso}}({}^{31}P)=4.6$ ppm is assigned to struvite, i.e., ${\mathrm{NH}}_{4}{\mathrm{MgPO}}_{4}\cdot 6H_{2}O$ (Bak et al., 2000). The correlation centered at $\delta_{\mathrm{iso}}({}^{31}P)=4.6\;\mathrm{ppm}↔\delta_{\mathrm{iso}}({}^{1}H)=8.7\;\mathrm{ppm}$ (ammonium groups) is attributed to ${\mathrm{PO}}_{4}^{3-}/{\mathrm{NH}}_{4}^{+}$ . The correlation centered at $\delta_{\mathrm{iso}}({}^{31}P)=4.6\;\mathrm{ppm}↔\delta_{\mathrm{iso}}({}^{1}H)=5.7\;\mathrm{ppm}$ concerns water molecules. It is interesting to note that the amount of struvite is extremely small (almost absent in the ${}^{31}P$ MAS NMR spectrum of KS4 and KS5; Fig. 9a and b). (ii) The resonance at $\delta_{\mathrm{iso}}({}^{31}P)=0.3$ ppm may be attributed to phosphates in phospholipids (in this case, $\delta_{\mathrm{iso}}({}^{31}P)$ is in the ∼1 to -1 ppm range). However, correlations with $\delta_{\mathrm{iso}}({}^{1}H)<3$ ppm are almost absent (such resonances should be characteristic for long alkyl chains in phospholipids). Consequently, we assign the ${}^{31}P$ resonance to inorganic (hydrated) orthophosphates. (iii) $\delta_{\mathrm{iso}}({}^{31}P)∼2.7$ ppm could be potentially assigned to amorphous calcium phosphate with a rather small (rather unusual) level of protonation (this resonance is underestimated in the CP MAS experiment; Fig. 9a). (iv) $\delta_{\mathrm{iso}}({}^{31}P)\ll 0$ ppm resonances are assigned to pyro- and/or polyphosphates. The ${}^{13}C$ CP MAS NMR spectra and ${}^{31}P$ (MAS and CP MAS) NMR spectra of all samples studied here are given in Figs. A3 and A4, respectively.

## 6 Syntheses of hydrated calcium oxalate, kidney stones samples and NMR methods

*Synthesis*. Calcium chloride ( ${\mathrm{CaCl}}_{2}$ ) and sodium oxalate ( ${\mathrm{Na}}_{2}C_{2}O_{4}$ ) were purchased from Sigma-Aldrich and used as received. All syntheses were carried out using distilled water. For COM, at 40 ${}^{\circ }$ C, equimolar aqueous solutions of ${\mathrm{Na}}_{2}C_{2}O_{4}$ and ${\mathrm{CaCl}}_{2}$ (0.1 mol. L ${}^{-1}$ ) were added simultaneously, dropwise, in a few milliliters of water under magnetic stirring. The mixture was left mixing under these conditions during 2 h before filtration and was then washed with cold water before drying under air. For COD, a ${\mathrm{Na}}_{2}C_{2}O_{4}$ aqueous solution (0.1 mol. L ${}^{-1}$ ) and a ${\mathrm{CaCl}}_{2}$ solution (1.0 mol. L ${}^{-1}$ , Ca/Ox =10 ) were prepared the day prior to the reaction and stored between 2–6 ${}^{\circ }$ C overnight. The solution of ${\mathrm{Na}}_{2}C_{2}O_{4}$ was added dropwise to the ${\mathrm{CaCl}}_{2}$ solution in an ice bath ( T<7 ${}^{\circ }$ C) under magnetic stirring. The mixture was left under stirring for 15 min before filtration and was then washed with cold water before drying under air. For COT, in an ice bath, two equimolar (0.001 mol. L ${}^{-1}$ ) aqueous solutions of ${\mathrm{Na}}_{2}C_{2}O_{4}$ and ${\mathrm{CaCl}}_{2}$ were slowly added simultaneously, dropwise, in a few milliliters of water under vigorous magnetic stirring. The mixture was left under stirring for 15 min before filtration and was then washed with cold water before drying under air. All COM, COD and COT samples were obtained as white fine powders. COD and COT were rapidly stored between 2–6 ${}^{\circ }$ C, while COM could be stored at ambient temperature.

*Kidney stones*. The samples were provided by Michel Daudon (Tenon Hospital, Paris, France). The choice of the diameter of the used NMR rotor was dictated by the initial size of the KSs and the implemented experiments. In the case of large KSs, smaller pieces were studied as powders by NMR.

*NMR methods*. Warning: the COM structure is highly sensitive to temperature variations ( ≥15 ${}^{\circ }$ C). The lowest MAS frequencies have to be implemented for all investigated nuclei and active regulation of the sample temperature (Bruker BCU-Xtreme cooling unit). Most of the ${}^{1}H$ MAS and DUMBO MAS NMR spectra presented in Fig. 1 were obtained at 700 MHz (Bruker AVANCE III spectrometer), using a 2.5 mm Bruker MAS probe spinning the sample at 12 kHz (20 to 40 scans; 10 s recycle delay for quantitative measurements; 10 ${}^{\circ }$ C in temperature; $t_{90{}^{\circ }}({}^{1}H)=3.0$ µs ; 24 µs duration of the shape length at 113 kHz radio frequency (RF) field). The DUMBO experiment was first set up with glycine as a test sample (including the scaling of the isotropic chemical shift) and then optimized for each compound. Some ${}^{1}H$ MAS NMR spectra were obtained at 850 MHz, using a 1 mm JEOL MAS probe (spinning the sample up to 79 kHz; four scans; 3 s recycle delay; $t_{90{}^{\circ }}({}^{1}H)=1.70$ µs ). Synchronized Hahn echoes (Fig. 4) were performed at 700 MHz, using a 2.5 mm Bruker MAS probe spinning the sample at 30 kHz (64 scans; 5 s recycle delay; $t_{90{}^{\circ }}({}^{1}H)=2.8$ µs ; no active regulation of the temperature in order to increase local dynamics; the increase in temperature is estimated to ∼40 ${}^{\circ }$ C). The ${}^{1}H{-}^{1}H$ DQF COSY MAS NMR experiment (Fig. 5) was performed at 700 MHz, using a 2.5 mm Bruker MAS probe at 30 kHz (32 scans; 2 s recycle delay; $t_{90{}^{\circ }}({}^{1}H)=2.8$ µs ; 256 increments in $t_{1}$ dimension; no active regulation of the temperature in order to increase local dynamics and magnitude mode). The ${}^{1}H{-}^{1}H$ SQ-DQ BABA MAS NMR experiment (Fig. 5) was performed at 850 MHz, using a 1 mm JEOL MAS NMR probe spinning the sample at 79 kHz (16 scans; 3 s recycle delay; $t_{90{}^{\circ }}({}^{1}H)=1.70$ µs ; two BABA loops, 426 increments in $t_{1}$ dimension and no active regulation of the temperature). All ${}^{1}H$ NMR spectra were referenced using adamantane (1.85 ppm) as a secondary reference. All natural abundance ${}^{43}\mathrm{Ca}$ NMR spectra (Fig. 6) were obtained at 850 MHz (Bruker AVANCE III spectrometer), using a 7 mm low- γ Bruker MAS single channel NMR probe spinning the sample at 3 to 5 kHz. A DFS (double frequency sweep; Iuga et al., 2000) enhancement scheme, followed by a 90 ${}^{\circ }$ selective pulse of 1.5 µs , was used (DFS pulse length of 2 ms, RF ∼8 kHz and a convergence sweep from 400 to 50 kHz; 5600 to 18 000 scans; 0.8 s recycle delay). All ${}^{43}\mathrm{Ca}$ chemical shifts were referenced at 0.0 ppm to a 1.0 mol. L ${}^{-1}$ aqueous solution of ${\mathrm{CaCl}}_{2}$ (Gervais et al., 2008). The ${}^{1}H{-}^{13}C$ RAMP (ramped amplitude) CP MAS experiments (Fig. 7) were obtained at 700 MHz (Bruker AVANCE III spectrometer) using a 2.5 mm Bruker MAS double resonance NMR probe spinning the sample at 5 kHz (600 to 1200 scans; 3 s recycle delay; $t_{90{}^{\circ }}({}^{1}H)=3.1$ µs ; 2 to 8 ms contact time). The ${}^{13}C$ MAS NMR spectra presented in Fig. 8 were obtained at 300 MHz (Bruker AVANCE III spectrometer) using a 7 mm Bruker MAS double resonance NMR probe spinning the sample at 5 kHz (328 scans; 3 s recycle delay; $t_{90{}^{\circ }}({}^{1}H)=5.2$ µs ; 0.8 and 9.0 ms contact time; refocused INEPT MAS: 6000 scans; 3 s recycle delay; 5.2 and 3.2 µs π/2 pulse on ${}^{1}H$ and ${}^{13}C$ respectively; no active regulation of the temperature). All ${}^{13}C$ NMR spectra were referenced using adamantane (38.48 ppm) as a secondary reference. ${}^{31}P$ 1D and 2D NMR spectra presented in Fig. 9 were obtained at 700 MHz (Bruker AVANCE III spectrometer) using a 2.5 mm Bruker MAS double resonance NMR probe spinning the sample at 30 kHz ( ≈4000 scans for high-power $\left\{{}^{1}H\right\}$ decoupling experiments and ≈3700 for CP MAS experiments; recycle delay: 10 s for high-power $\left\{{}^{1}H\right\}$ decoupling experiments; 30 ${}^{\circ }$ flip angle; 3 s for CP MAS experiments; $t_{90{}^{\circ }}({}^{1}H)=2.0$ µs ; 5.0 ms contact time for CP MAS experiments). For the ${}^{1}H{-}^{31}P$ HETCOR RAMP CP MAS experiment, the number of scans was 400, the recycle delay was 3 s, $t_{90{}^{\circ }}({}^{1}H)=2.0$ µs , the contact time was 5.0 ms, with 96 increments in $t_{1}$ dimension, and there was an active regulation of the temperature at -20 ${}^{\circ }$ C.

*Relaxation of crystallographic structures*. Starting from the crystallographic data, COM (Daudon et al., 2009), COD (Tazzoli and Domeneghetti, 1980) and COT (Basso et al., 1997) structures were relaxed at DFT level. The unit cell parameters and the atomic positions were optimized, as previously described for COM (Colas et al., 2013). The Vienna Ab initio Simulation Package (VASP) was used (Kresse and Hafner, 1993, 1994; Kresse and Furthmüller, 1996). The corresponding crystallographic information files (CIFs) are available upon request.

## 7 Conclusions and perspectives

This study has demonstrated that the solid-state NMR technique offers a complementary characterization approach for the study of kidney stones and related synthetic model systems. The ${}^{1}H$ DUMBO MAS NMR technique provides unambiguous identification of the different calcium oxalate hydrate phases. This experiment is a rapid-measurement technique which can be easily adapted to yield semi-quantitative data. For the first time, the natural abundance ${}^{43}\mathrm{Ca}$ MAS NMR data from the three calcium oxalate hydrate phases have been presented together; these data exhibited sufficient signal to noise to facilitate a complete structural interpretation in agreement with crystallographic data. The extension of this approach to the study of KSs was attempted, showing that a real signal could be measured but with relatively limited discrimination between the different KSs samples. The deconvolution of the ${}^{1}H$ and ${}^{13}C$ MAS NMR data into assigned subspectra aided the interpretation of the data describing the whole system, thus demonstrating that KSs materials are usually a complex association of organic and inorganic components. Additional ${}^{31}P$ MAS NMR studies provided further insight into the composition of the low-level phosphates which are ubiquitous and difficult to characterize in KSs. The development of solid-state NMR, in combination with modern computational DFT and machine learning approaches, would be able to characterize the complex heterogeneous biomaterials such as KSs without ambiguity (Tielens et al., 2021). As part of on-going studies building on the observations here, systematic NMR studies of a large range of KSs from the Tenon Hospital's collection is being undertaken to develop new diagnosis NMR approaches that could impact on developing novel treatments.

## Data Availability

All the data are shown in all the figures of the paper. The CIFs of COM, COD and COT structures are available upon request from the corresponding author.

## Appendix A

**Table A1:** All CIFs are available upon request for COM, COD and COT. **(a)** Selected $O-\underline{H}\ldots \underline{O}$ distances and the number of H…O contacts with $O-\underline{H}\ldots \underline{O}\leq 3$ Å (italic) for COM. The atomic positions were optimized at the DFT level. **(b)** Selected $O-\underline{H}\ldots \underline{O}$ distances and the number of H…O contacts with $O-\underline{H}\ldots \underline{O}\leq 3$ Å (italic) for COD. The atomic positions were optimized at the DFT level. In order to take into account the distribution of the zeolitic water molecules, a model corresponding to ${\mathrm{CaC}}_{2}O_{4}\cdot (2+0.375)H_{2}O$ was first calculated (see below), i.e., ${\mathrm{Ca}}_{8}C_{16}O_{32}(H_{2}O{)}_{16}(H_{2}O{)}_{3}$ . The less rigid molecules are highlighted in bold. The first four molecules are structural. The last three molecules are zeolitic. **(c)** Selected $O-\underline{H}\ldots \underline{O}$ distances and the number of H…O contacts with $O-\underline{H}\ldots \underline{O}\leq 3$ Å (bold italic) for COT. The atomic positions were optimized at the DFT level.

(a)	H11	O100	1.00849	H12	O100	0.98369	H21	O200	0.99077	H22	O200	0.98971									
	H11	H12	1.57350	H12	H11	1.57350	H21	H22	1.60332	H22	H21	1.60332									
	*H11*	*O8*	1.64743	*H12*	*O5*	*1.95727*	*H21*	*O5*	*1.75421*	*H22*	*O100*	*1.84259*									
	H11	C4	2.32231	H12	H22	2.34911	H21	C3	2.41467	H22	H12	2.34911									
	H11	H22	2.38986	H12	C3	2.43432	*H21*	*O6*	*2.59359*	H22	H11	2.38986									
	*H11*	*O7*	2.54004	*H12*	*O1*	*2.82575*	H21	Ca1	2.91456												
	H11	Ca2	2.97148	H12	Ca2	2.90392	H21	H12	2.92563												
				H12	H21	2.92563															
				*H12*	*O8*	*2.97218*															
(b)	H1	O33	0.98706	H2	O34	0.98980	H3	O34	0.98182	H4	O33	0.99442	H5	O35	0.98170	H6	O36	0.98695	H7	O36	0.98579
	H1	H4	1.59565	H2	H3	1.57549	H3	H2	1.57549	H4	H1	1.59565	H5	H8	1.61227	H6	H7	1.58205	H7	H6	1.58205
	*H1*	*O26*	*1.91539*	*H2*	*O25*	*1.82678*	*H3*	*O28*	*2.04985*	*H4*	*O27*	1.*75128*	*H5*	*O30*	*1.85900*	*H6*	*O29*	*1.87561*	*H7*	*O32*	*1.91845*
	H1	H17	2.11120	H2	H18	2.12287	H3	H19	2.22917	H4	H38	1.97874	H5	H21	*2.15627*	H6	H22	2.15494	H7	H23	2.15003
	H1	H38	2.35789	H2	H37	2.24223	H3	H37	2.66372	H4	H20	2.04373	*H5*	*O14*	*2.75907*	H6	H37	2.31981	H7	H22	2.69686
	H1	H20	2.69799	H2	H19	2.67477	H3	H18	2.70041	H4	H17	2.52979	H5	C10	2.81281	H6	H23	2.64503	*H7*	*O16*	*2.78683*
	*H1*	*O44*	*2.73922*	H2	H8	2.74763	*H3*	*O12*	*2.81150*	*H4*	*O44*	*2.63337*	H5	*H24*	*2.83299*	*H6*	*O42*	*2.77692*	*H7*	*O42*	*2.79486*
	*H1*	*O10*	*2.79429*	*H2*	*O9*	*2.75952*	*H3*	*O43*	*2.84290*	H4	C13	2.68644	*H5*	*O41*	*2.88697*	*H6*	*O13*	*2.79229*	H7	H37	2.81901
	H1	C16	2.86535	H2	C15	2.77043	H3	H8	2.86354	*H4*	*O11*	*2.84953*	H5	Ca3	2.93317	H6	C9	2.81462	H7	C12	2.85808
	H1	Ca1	2.99269	*H2*	*O43*	*2.77691*	H3	C14	2.95471	*H4*	*O51*	*2.90084*				H6	Ca4	2.92805	H7	Ca4	2.95463
				*H2*	*O51*	*2.78975*	H3	Ca2	2.98530	*H4*	*O4*	*2.93927*				*H6*	*O6*	*2.99753*			
				H2	Ca2	2.93267				H4	Ca1	2.96741									
	H8	O35	1.00238	H9	O37	0.98455	H10	O38	0.98386	H11	O38	0.98764	H12	O37	0.98525	H13	O39	0.98603	H14	O40	0.98212
	H8	H5	1.61227	H9	H12	1.57848	H10	H11	1.59461	H11	H10	1.59461	H12	H9	1.57848	H13	H16	1.60309	H14	H15	1.57384
	*H8*	*O51*	*1.66198*	*H9*	*O18*	*1.89267*	*H10*	*O17*	*1.85718*	*H11*	*O50*	*1.85667*	*H12*	*O19*	*1.84639*	*H13*	*O50*	*1.83852*	*H14*	*O21*	*2.11357*
	H8	H38	1.99179	H9	H25	2.14808	H10	H26	2.12211	H11	H36	2.17741	H12	H28	2.07478	H13	H36	2.35234	H14	H30	2.23119
	H8	H37	2.04976	H9	H36	2.36458	H10	H33	2.34664	H11	H13	2.40635	H12	H34	2.50717	H13	H11	2.40635	H14	H36	2.65940
	H8	H2	2.74763	H9	H28	2.64453	H10	H27	2.65917	H11	H35	2.60426	H12	H25	2.62744	H13	H35	2.56253	H14	H31	2.70447
	*H8*	*O15*	*2.84946*	*H9*	*O48*	*2.77037*	*H10*	*O47*	*2.76488*	*H11*	*O20*	*2.76299*	*H12*	*O48*	*2.71565*	*H13*	*O22*	*2.81506*	*H14*	*O5*	*2.79807*
	H8	H3	2.86354	*H9*	*O2*	*2.78591*	H10	C7	2.80358	*H11*	*O39*	*2.81858*	*H12*	*O3*	*2.76892*	H13	H29	2.85749	*H14*	*O46*	*2.83725*
	H8	H24	2.93412	H9	C8	2.84076	*H10*	*O1*	*2.88960*	*H11*	*O4*	*2.82251*	H12	C5	2.83943	*H13*	*O6*	*2.93190*	*H14*	*O38*	*2.89969*
	*H8*	*O31*	*2.96498*	H9	Ca5	2.93707	H10	O39	2.92353	H11	H27	2.85030	H12	Ca5	2.95153				H14	H33	2.94927
	*H8*	*O34*	*2.99827*	*H9*	*O50*	*2.98631*	H10	Ca6	2.98407				H12	H36	2.99920						
	H15	O40	0.99513	H16	O39	0.98321	**H17**	**O44**	**0.98418**	**H18**	**O43**	**0.98306**	**H19**	**O43**	**0.98845**	**H20**	**O44**	**0.98141**	**H21**	**O41**	**0.98524**
	H15	H14	1.57384	H16	H13	1.60309	**H17**	**H20**	**1.58638**	**H18**	**H19**	**1.58312**	**H19**	**H18**	**1.58312**	**H20**	**H17**	**1.58638**	**H21**	**H24**	**1.59032**
	*H15*	*O24*	*1.77827*	*H16*	*O23*	*1.89139*	***H17***	***O26***	***1.88660***	***H18***	***O25***	***1.90254***	***H19***	***O28***	***1.83208***	***H20***	***O27***	***1.97101***	***H21***	***O30***	***1.84549***
	H15	H31	2.12464	H16	H32	2.16066	**H17**	**H1**	**2.11120**	**H18**	**H2**	**2.12287**	**H19**	**H3**	**2.22917**	**H20**	**H4**	**2.04373**	**H21**	**H5**	**2.15627**
	H15	H36	2.19988	H16	H29	2.69091	**H17**	**C16**	**2.50781**	**H18**	**C15**	**2.52659**	**H19**	**C14**	**2.48695**	**H20**	**C13**	**2.60434**	**H21**	**C10**	**2.45463**
	H15	H30	2.67389	H16	C3	2.79378	**H17**	**H4**	**2.52979**	**H18**	**H3**	**2.70041**	**H19**	**H2**	**2.67477**	**H20**	**H1**	**2.69799**	***H21***	***O35***	***2.74039***
	H15	C4	2.70528	*H16*	*O45*	*2.80838*	***H17***	***O33***	***2.71797***	***H18***	***O34***	***2.78743***	***H19***	***O34***	***2.82331***	***H20***	***O33***	***2.77138***			
	*H15*	*O8*	*2.75027*	*H16*	*O7*	*2.82588*	***H17***	***O1***	***2.96592***												
	*H15*	*O46*	*2.77041*	H16	H33	2.95661															
	*H15*	*O50*	*2.91170*	H16	Ca7	2.98301															

**Table A1:** Continued.

	**H22**	**O42**	**0.98588**	**H23**	**O42**	**0.98660**	**H24**	**O41**	**0.99754**	H25	O48	0.98498	H26	O47	0.98243	H27	O47	0.99450	H28	O48	0.98088
	**H22**	**H23**	**1.58163**	**H23**	**H22**	**1.58163**	**H24**	**H21**	**1.59032**	H25	H28	1.57886	H26	H27	1.58899	H27	H26	1.58899	H28	H25	1.57886
	***H22***	***O29***	***1.91449***	***H23***	***O32***	***1.87699***	***H24***	***O31***	***1.70417***	*H25*	*O18*	*1.89272*	*H26*	*O17*	*1.88627*	*H27*	*O20*	*1.73992*	*H28*	*O19*	*1.95113*
	**H22**	**H6**	**2.15494**	**H23**	**H7**	**2.15003**	**H24**	**C11**	**2.43596**	H25	H9	2.14808	H26	H10	2.12211	H27	C6	2.43921	H28	H12	2.07478
	**H22**	**C9**	**2.54313**	**H23**	**C12**	**2.50035**	**H24**	**H5**	**2.83299**	H25	C8	2.53078	H26	C7	2.49549	H27	H10	2.65917	H28	C5	2.52406
	**H22**	**H7**	**2.69686**	**H23**	**H6**	**2.64503**	***H24***	***O35***	***2.93124***	H25	H12	2.62744	*H26*	*O7*	*2.69101*	*H27*	*O6*	*2.68011*	H28	H9	2.64453
	***H22***	***O36***	***2.82367***	***H23***	***O36***	***2.79218***	**H24**	**H8**	**2.93412**	*H25*	*O8*	*2.73239*	H26	Ca7	2.86542	H27	Ca7	2.83851	*H28*	*O5*	*2.71256*
				***H23***	***O7***	***2.97205***				*H25*	*O37*	*2.78268*	*H26*	*O38*	*2.88787*	H27	H11	2.85030	*H28*	*O37*	*2.74612*
										H25	Ca8	2.87171				*H27*	*O38*	*2.98955*	H28	Ca8	2.91374
	H29	O45	0.99748	H30	O46	0.99081	H31	O46	0.98265	H32	O45	0.98460									
	H29	H32	1.58925	H30	H31	1.58606	H31	H30	1.58606	H32	H29	1.58925									
	*H29*	*O22*	*1.72144*	*H30*	*O21*	*1.80278*	*H31*	*O24*	*1.92821*	*H32*	*O23*	*1.85294*									
	H29	C2	2.42196	H30	H14	2.23119	H31	H15	2.12464	H32	H16	2.16066									
	*H29*	*O2*	*2.65935*	H30	C1	2.47815	H31	C4	2.55193	H32	C3	2.49151									
	H29	H16	2.69091	H30	H15	2.67389	H31	H14	2.70447	*H32*	*O3*	*2.71237*									
	H29	Ca5	2.83343	*H30*	*O1*	*2.71669*	*H31*	*O4*	*2.74962*	*H32*	*O39*	*2.81984*									
	H29	H13	2.85749	*H30*	*O40*	*2.85346*	*H31*	*O40*	*2.82082*	H32	Ca5	2.85738									
	*H29*	*O39*	*2.87558*	H30	Ca6	2.85690	H31	Ca6	2.88812												
				*H30*	*O14*	*2.87981*															
	**H33**	**O49**	**0.97686**	**H34**	**O49**	**0.97560**	**H35**	**O50**	**0.98605**	**H36**	**O50**	**0.97905**	**H37**	**O51**	**0.97973**	**H38**	**O51**	**0.99205**			
	**H33**	**H34**	**1.55468**	**H34**	**H33**	**1.55468**	**H35**	**H36**	**1.58568**	**H36**	**H35**	**1.58568**	**H37**	**H38**	**1.55005**	**H38**	**H37**	**1.55005**			
	**H33**	**H10**	**2.34664**	***H34***	***O19***	***2.39218***	***H35***	***O49***	***1.84598***	***H36***	***O40***	***2.14165***	**H37**	**H8**	**2.04976**	***H38***	***O33***	***1.77829***			
	**H33**	**H35**	**2.58196**	**H34**	**H12**	**2.50717**	**H35**	**H34**	**2.53067**	**H36**	**H11**	**2.17741**	***H37***	***O34***	***2.20072***	**H38**	**H4**	**1.97874**			
	***H33***	***O38***	***2.66257***	**H34**	**H35**	**2.53067**	**H35**	**H13**	**2.56253**	**H36**	**H15**	**2.19988**	**H37**	**H2**	**2.24223**	**H38**	**H8**	**1.99179**			
	***H33***	***O17***	***2.72932***	**H34**	**C5**	**2.65374**	**H35**	**H33**	**2.58196**	**H36**	**H13**	**2.35234**	**H37**	**H6**	**2.31981**	**H38**	**H1**	**2.35789**			
	**H33**	**H14**	**2.94927**	**H34**	**C3**	**2.91240**	**H35**	**H11**	**2.60426**	**H36**	**H9**	**2.36458**	***H37***	***O36***	***2.45695***						
	**H33**	**H16**	**2.95661**	***H34***	***O3***	***2.96977***				***H36***	***O37***	***2.61085***	**H37**	**H3**	**2.66372**						
	**H33**	**C7**	**2.98511**							**H36**	**H14**	**2.65940**	**H37**	**H7**	**2.81901**						
										***H36***	***O38***	***2.89925***									
										**H36**	**H12**	**2.99920**									
(c)	H1	O6	0.98194	H2	O6	1.01013	H3	O7	0.98972	H4	O5	1.00726	H5	O5	0.99237	H6	O7	0.98705			
	H1	H2	1.62553	H2	H1	1.62553	H3	H6	1.57438	H4	H5	1.61032	H5	H4	1.61032	H6	H3	1.57438			
	*H1*	*O3*	*1.97834*	*H2*	*O1*	*1.66830*	*H3*	*O6*	*1.83746*	*H4*	*O2*	*1.67978*	*H5*	*O3*	*1.81868*	*H6*	*O5*	*1.95753*			
	H1	H3	2.09660	H2	H3	2.15532	H3	H1	2.09660	H4	H6	2.08633	H5	H1	2.37604	H6	H4	2.08633			
	H1	H5	2.37604	H2	C1	2.61465	H3	H2	2.15532	H4	C1	2.60506	H5	C2	2.54822	H6	H5	2.66412			
	H1	C2	2.73894	H2	H4	2.75756	H3	H1	2.82312	H4	H2	2.75756	H5	H6	2.66412	*H6*	* O2*	*2.80700*			
	H1	H3	2.82312	*H2*	*O4*	*2.79380*	*H3*	*O3*	*2.87563*	*H4*	*O7*	*2.83920*	*H5*	*O4*	*2.77186*	H6	H4	*2.84626*			
	*H1*	*O7*	*2.84845*	*H2*	*O4*	*2.96096*	*H3*	*O6*	*2.96615*	H4	H6	2.84626	*H5*	*O2*	*2.84989*	*H6*	*O2*	*2.85413*			
	*H1*	*O7*	*2.91450*				H3	Ca1	2.98521	*H4*	*O7*	*2.85001*	H5	Ca1	2.91831						
	H1	H4	2.93242							H4	H1	2.93242									
	*H1*	*O5*	2.93253							*H4*	*O1*	*2.99648*									
	H1	H1	2.95802																		
